# Comparative antimicrobial potential of silver and zinc oxide nanoparticles versus chlorhexidine and tetracycline-based local drug delivery agents against periodontal pathogens: a systematic review

**DOI:** 10.1186/s11671-026-05027-1

**Published:** 2026-11-02

**Authors:** Shahabe Saquib Abullais, Prasad Dhadse, Mohan Kumar P

**Affiliations:** 1 Department of Periodontology, Sharad Pawar Dental College, Adani Institute of Higher Education and Research, Sawangi, Wardha, 442107 India; 2 Department of Periodontics and Implantology, Vishnu Dental College, Vishnupur, West Godavari, 534202 Bhimavaram, Andhra Pradesh India

**Keywords:** Antimicrobial resistance, Biofilm, Local drug delivery, Nanotechnology, Periodontitis, Periodontal Pathogens

## Abstract

The objective of this study is to compare the antimicrobial effectiveness of Silver (Ag) and Zinc oxide (ZnO) nanoparticles with both Chlorhexidine and Tetracycline used as TDS’s through *in-vitro* studies.The study employed a systematic review following the PRISMA guidelines to identify and select studies published up to 2025 from the electronic databases PubMed, Scopus, Cochrane, and WEB of Science. No search limit was set. Studies selected reported in vitro comparisons between Ag and/or ZnO nanomaterials and traditional antimicrobial drugs using oral microbial pathogens. Both assessors independently performed study selection, data extraction, and quality assessment using the Joanna Briggs Institute checklist. The primary outcomes were minimum inhibitory concentrations and zones of inhibition. Of the ten studies included in this analysis, none of the studies found Ag nanoparticles to be less effective than chlorhexidine against a range of microorganisms; however, Ag nanoparticles had significantly lower minimum inhibitory concentration (MIC) values than chlorhexidine and had a significantly larger zone of inhibition than chlorhexidine in most studies examined. Zinc oxide nanoparticles were found to have moderate, concentration-dependent antimicrobial activity, which tended to be comparable to chlorhexidine, but not to antibiotic tetracycline. Ag nanoparticle-antibiotic combinations exhibited a synergistic effect in seven of the studies reviewed. Few studies reported a risk of bias. Based on the included in vitro studies, Ag nanomaterials demonstrated promising direct antimicrobial activity against periodontal pathogens. However, owing to methodological heterogeneity and the absence of clinical evidence, definitive conclusions regarding their superiority or routine clinical use cannot yet be drawn. Further standardized biological, preclinical, and clinical investigations are warranted.

## Introduction

Chronic inflammatory conditions associated with the tooth’s supporting structures, such as gingiva, periodontal ligaments, and alveolar bone, are frequently affected by periodontal disease. Microbial dysbiosis, which occurs due to specific microflora in the subgingival biofilm, is responsible for initiating and perpetuating periodontal disease [1]. Pathogenic bacteria such as *Porphyromonas gingivalis* and *Aggregatibacter actinomycetemcomitans*,and other anaerobic bacteria, play an essential role in the progression of periodontal disease within this biofilm [2]. To accomplish effective periodontal disease management, pathogenic bacteria must first be reduced and the formation of biofilm must be controlled [3]. Antimicrobial drugs are often used as adjunctive agents in conjunction with mechanical cleaning methods (debridement) for the treatment of periodontal disease [3, 4].

The use of Local drug delivery (LDD) systems that are loaded with antimicrobial agents (e.g., chlorhexidine and tetracycline class antibiotics) has become widespread within the periodontal therapeutic community [5]. Unlike the traditional mechanism of delivering these drugs through the bloodstream, LDD systems allow for greater local concentrations of drug to be delivered directly into periodontal pockets while minimizing systemic exposure [5, 6]. Although researchers have documented the rapid onset of clinical efficacy for both chlorhexidine and tetracycline LDDs, the widespread and long-term use of these traditional antimicrobial therapies presents three significant problems: development of resistance to antibiotics; cytotoxicity of the antimicrobial agent(s) on resident host tissue; and reduction of antibiotic effectiveness against resistant microbial strains. Due to these adverse effects associated with traditional antimicrobial therapies, alternative antimicrobial strategies are warranted within the context of periodontal disease treatment [7–9].

Despite substantial advances in non-surgical periodontal therapy and adjunctive local drug delivery systems, the management of periodontitis remains challenging in many clinical situations. Periodontitis continues to be one of the most prevalent chronic inflammatory diseases worldwide and is a major cause of tooth loss in adults. Mechanical debridement alone may not completely eliminate pathogenic microorganisms from deep periodontal pockets, furcation areas, and other anatomically complex sites where mature biofilms persist. Although chlorhexidine and tetracycline-based local drug delivery systems improve microbial control, concerns remain regarding bacterial resistance, cytotoxicity, limited penetration into established biofilms, and variability in long-term clinical outcomes. Consequently, there is an ongoing need to investigate alternative antimicrobial strategies capable of disrupting periodontopathogenic biofilms more effectively while maintaining favorable biocompatibility. Nanomaterials, particularly silver (Ag) and Zinc oxide (ZnO) nanoparticles, have therefore emerged as promising candidates because of their broad-spectrum antimicrobial activity, biofilm-disrupting properties, and lower propensity for inducing microbial resistance.

Nanotechnology has emerged as a transformative approach in modern dentistry owing to its ability to improve the prevention, diagnosis, and treatment of oral diseases. Nanomaterials have been incorporated into a wide range of dental applications, including restorative materials, implant surface modifications, tissue engineering, drug delivery systems, antimicrobial coatings, and preventive oral healthcare products. Their unique physicochemical properties, such as high surface-area-to-volume ratio, enhanced reactivity, and the ability to interact with microbial biofilms at the nanoscale, make them particularly attractive for controlling oral pathogens. These advantages have stimulated increasing research into nanotechnology-based antimicrobial strategies aimed at improving the management of biofilm-associated diseases, including periodontitis.

The present review specifically focuses on silver (Ag) and Zinc oxide (ZnO) nanoparticles because these nanomaterials have demonstrated promising antimicrobial activity against periodontal pathogens and have been extensively investigated as potential alternatives or adjuncts to conventional local antimicrobial agents.

Nanotechnology has developed to be a promising new tool for biological and dental research in recent years [10]. Nanoparticles like Ag and ZnO have shown to be very strong antimicrobials due to their high surface area and high reactivity, and the ability to disrupt both the cell membranes and metabolic processes of microorganisms [10–12]. Several *in-vitro* studies have demonstrated that Ag and ZnO nanoparticles possess promising antimicrobial activity against periodontal pathogens, suggesting their potential as alternative or adjunctive antimicrobial agents in periodontal therapy. However, these findings should be interpreted with caution. Despite their broad-spectrum antimicrobial and biofilm-disrupting properties, concerns remain regarding their potential cytotoxic, genotoxic, mutagenic, and dose-dependent biological effects on host cells and tissues. Furthermore, differences in nanoparticle size, concentration, synthesis methods, and experimental conditions contribute to variability in reported outcomes. Consequently, although Ag and ZnO nanoparticles represent promising candidates for periodontal applications, further standardized in vitro investigations, biocompatibility assessments, and well-designed clinical studies are required before their routine clinical use can be recommended [13–15].

Several studies have looked at the antimicrobial effect of Ag and ZnO nanomaterials in isolation; however, results are inconsistent and vary according to factors including the size, concentration, method of synthesis, and type of microorganisms being tested [16–18]. There are also a few studies that have provided consolidated data on the antimicrobial effectiveness of these types of nanomaterials compared with broadly used conventional treatments such as chlorhexidine and tetracycline based local drug delivery systems [18]. Currently, little comprehensive *in-vitro* evidence exists that compares these agents against pathogens that cause periodontal disease [19].

Although numerous *in-vitro* studies have investigated the antimicrobial activity of Ag and ZnO nanoparticles against periodontal pathogens, the available evidence remains heterogeneous because of differences in nanoparticle characteristics, synthesis methods, concentrations, microbial strains, and outcome assessment techniques. Consequently, the comparative effectiveness of these nanomaterials relative to conventional local antimicrobial agents, such as chlorhexidine and tetracycline, remains uncertain. To date, no systematic review has comprehensively synthesized this evidence to provide a direct comparison of these nanomaterials with established local drug delivery agents used in periodontal therapy.

Therefore, the present systematic review was conducted to critically evaluate and synthesize the available in vitro evidence comparing the antimicrobial efficacy of Ag and ZnO nanoparticles with chlorhexidine and tetracycline against periodontal pathogens. The comparison was restricted to chlorhexidine and tetracycline-based local drug delivery systems because they are among the most extensively studied and clinically established adjunctive antimicrobial agents in periodontal therapy. They represent the current standard local antimicrobial treatments, making them appropriate and clinically relevant comparators for evaluating the antimicrobial potential of Silver and Zinc oxide nanoparticles. Restricting the comparators also reduced clinical heterogeneity and enabled a more focused and meaningful synthesis of the available evidence.

Unlike previous narrative discussions of nanomaterials, this review systematically appraises and compares the available evidence using a structured methodology, thereby providing a clearer understanding of their relative antimicrobial performance. By identifying methodological limitations, inconsistencies, and research gaps, this review may help guide the design of future experimental studies, promote standardization of in vitro research, and support the development of safer and more effective nanotechnology-based antimicrobial strategies for periodontal therapy. The review addresses the following research question: In *in-vitro* studies evaluating periodontal pathogens, how does the antimicrobial activity of Ag and ZnO nanoparticles compare with that of chlorhexidine and tetracycline-based local antimicrobial agents?

## Methodology

### Study design and reporting guidelines

The systematic review was conducted and reported in accordance with the Preferred Reporting Items for Systematic Reviews and Meta-Analyses (PRISMA) 2020 Statement [20]. The review protocol was prospectively registered in PROSPERO on 27^th^ January 2026 (Registration No.ID: CRD420261294568) to enhance methodological transparency and prespecify the review methods. Although PROSPERO primarily focuses on health-related systematic reviews, the registration was intended to ensure transparent reporting. We acknowledge that the Open Science Framework (OSF) is increasingly recommended for systematic reviews of in vitro studies and may be a more suitable registration platform for future reviews of this nature.

### Eligibility criteria

The systematic review’s eligibility requirements were determined through the use of the PICOS framework, using population (P) Periodontopathogenic microorganisms included in this review were *Porphyromonas gingivalis, Aggregatibacter actinomycetemcomitans, Tannerella forsythia, Treponema denticola, Fusobacterium nucleatum, Prevotella intermedia, Prevotellanigrescens, Campylobacter rectus, Parvimonas micra* (formerly *Peptostreptococcus micros), Eikenellacorrodens, Capnocytophaga spp., Enterococcus faecalis*, and other periodontal microorganisms specifically evaluated in the included in vitro studies. The intervention (I) was made up of Ag and ZnO nanoparticles (i.e. nanomaterials). Ag NPs and ZnO NPs were considered as separate nanoparticle interventions, and each was compared independently with conventional antimicrobial agents, including chlorhexidine and tetracycline-based local drug delivery systems, where applicable.The comparison (C) consisted of conventional antimicrobial drugs, e.g. chlorhexidine and/or tetracycline local drug delivery systems. Outcome (O): The primary outcome was antimicrobial activity against periodontal pathogens assessed using both planktonic and biofilm-based experimental methods. Planktonic assays included minimum inhibitory concentration (MIC), minimum bactericidal concentration (MBC), and zone of inhibition. Biofilm-related outcomes included biofilm inhibition, colony-forming unit (CFU) enumeration, bacterial viability assays, metabolic activity assays (e.g., XTT or resazurin assays), biofilm biomass quantification (e.g., crystal violet staining), and other validated biofilm assessment techniques reported in the included studies.

### Inclusion and exclusion criteria

Through the use of specific inclusion and exclusion criteria, studies were selected for this review. The in vitro studies were reviewed, thus providing the basis for finding studies that evaluated the effectiveness of Ag and/or ZnO nanomaterials to combat bacteria known to cause and maintain periodontal disease. As well as looking at the existing literature for studies that compared These two nanomaterials compared with standard antimicrobial medications such as chlorhexidine or tetracycline for local drug delivery systems for their effectiveness in treating periodontal disease. Only studies specifically evaluating microorganisms associated with periodontal disease were considered eligible. Furthermore, only articles published in the English language were included in the review.

Studies were excluded if they are clinical studies, animal studies, review articles, or other secondary research publications. Studies that do not evaluate periodontal pathogens were also be excluded. In addition, studies that assess Ag or ZnO nanomaterials without comparing them with chlorhexidine or tetracycline-based agents were not be considered. Conference abstracts without full-text availability were also be excluded from the review.

### Information sources and search strategy

The electronic database search was conducted on 11 March 2026 and included all eligible studies published up to that date. Complete database-specific search strategies, including Boolean operators and MeSH terms, are provided separately. No filters for publication year or study design were applied during the search; however, only English-language publications were included because translation resources were unavailable, which is acknowledged as a potential source of language bias. Grey literature sources, including Google Scholar, OpenGrey, ProQuest Dissertations and Theses, conference proceedings, and reference lists, were also searched to identify unpublished studies. Unpublished studies were considered for inclusion if they met the predefined eligibility criteria and provided sufficient methodological details.

All literature retrieved from the electronic databases were exported into reference management software, and any duplicate articles were deleted before screening and reviewing literature identified as potential candidates for inclusion.All retrieved records were exported into Mendeley Reference Manager for reference management. Duplicate records were initially identified using the software’s automatic duplicate detection feature and were subsequently verified manually by two independent reviewers before removal to ensure accuracy.

Two different reviewers were independently screened the title and abstract of all identified literature using pre-established criteria for review eligibility; studies meeting the review eligibility requirements were chosen for full-text review; any disagreements between reviewers were resolved through discussion or were referred to a third-party reviewer. The inter-reviewer agreement was excellent (Cohen’s κ = 0.89).

All studies eligible to be included in the systematic review had their data extracted according to the guidelines set forth by Joanna Briggs Institute (JBI) [21]. The methodological quality and risk of bias of the literature selected for inclusion in the systematic review were evaluated using critical appraisal criteria set forth by JBI for *in-vitro* studies. The results from the selected studies were qualitatively summarized and synthesized.

### Search strategy

A comprehensive search of the literature was conducted to identify relevant studies regarding the antimicrobial activity of Ag and ZnO nanomaterials, and the comparative review with standard antimicrobial medications. The electronic databases of PubMed, Scopus, Cochrane, and WEB of Science, were searched for eligible studies through to the specified search date. In conducting the search for eligible studies, the appropriate keywords and search terms were included those used in relation to periodontal disease pathogens, nanomaterials, chlorhexidine, tetracycline, and antimicrobial activity. In addition to database searching, reference lists of the selected articles were manually screened to identify any additional relevant studies.

The search included studies published up to the year 2025. Keywords and Medical Subject Headings (MeSH) related to periodontal pathogens, nanomaterials, and antimicrobial agents were used.

### PubMed search strategy

(“Periodontal pathogens” OR “*Porphyromonas gingivalis*” OR “*Aggregatibacter actinomycetemcomitans*”) AND (“Silver nanoparticles” OR “Ag nanoparticles” OR “Zinc oxide nanoparticles” OR “ZnO nanoparticles”) AND (“Chlorhexidine” OR “Tetracycline” OR “Local drug delivery”) AND (“Antimicrobial activity” OR “MIC” OR “MBC” OR “Zone of inhibition”)

### Scopus search strategy

(TITLE-ABS-KEY(“periodontal pathogens” OR “*Porphyromonas gingivalis*” OR “*Aggregatibacter actinomycetemcomitans*” OR “periodontal bacteria”)) AND (TITLE-ABS-KEY(“silver nanoparticles” OR “Ag nanoparticles” OR “Zinc oxide nanoparticles” OR “ZnO nanoparticles” OR nanomaterials)) AND(TITLE-ABS-KEY(“chlorhexidine” OR “tetracycline” OR “local drug delivery”)) AND(TITLE-ABS-KEY(“antimicrobial activity” OR “minimum inhibitory concentration” OR MIC OR “minimum bactericidal concentration” OR MBC OR “zone of inhibition”))

### Web of science search strategy

TS = (“periodontal pathogens” OR “*Porphyromonas gingivalis*” OR “*Aggregatibacter actinomycetemcomitans*” OR “periodontal bacteria”) AND TS = (“silver nanoparticles” OR “Ag nanoparticles” OR “Zinc oxide nanoparticles” OR “ZnO nanoparticles” OR nanomaterials) AND TS = (“chlorhexidine” OR “tetracycline” OR “local drug delivery”) AND TS = (“antimicrobial activity” OR “minimum inhibitory concentration” OR MIC OR “minimum bactericidal concentration” OR MBC OR “zone of inhibition”)

### Cochrane Library (CENTRAL) search strategy

(“periodontal pathogens” OR “*Porphyromonas gingivalis*” OR “*Aggregatibacter actinomycetemcomitans*”) AND(“silver nanoparticles” OR “Ag nanoparticles” OR “Zinc oxide nanoparticles” OR “ZnO nanoparticles”) AND(“chlorhexidine” OR “tetracycline” OR “local drug delivery”) AND (“antimicrobial activity” OR “minimum inhibitory concentration” OR “minimum bactericidal concentration” OR “zone of inhibition”)

Manual searches of reference lists from selected articles were performed to identify additional relevant studies.

### Study selection

Two separate reviewers extracted data from the studies to be included via a common form. In cases of differing opinions on data extraction, discrepancies were resolved through discussions.In addition to electronic databases, grey literature sources (e.g., Google Scholar, OpenGrey, ProQuest Dissertations and Theses, conference proceedings, and reference lists of included studies) were searched to identify potentially eligible unpublished studies.

A quantitative meta-analysis was considered but was not performed because of substantial methodological heterogeneity among the included studies, including differences in nanoparticle characteristics, synthesis methods, concentrations, periodontal microorganisms, antimicrobial assays, and outcome reporting. Therefore, a narrative synthesis was considered the most appropriate approach.

### Data extraction

Data extraction was independently performed using a standardized form. The following information was extracted: author, year of publication, periodontal microorganisms evaluated, nanoparticle characteristics (type, size, morphology, synthesis method, concentration), comparator antimicrobial agent and concentration, antimicrobial assay employed, quantitative outcome measures, and principal findings. Antimicrobial assays included both planktonic methods (MIC, MBC, and zone of inhibition) and biofilm-based methods such as CFU enumeration, bacterial viability assays, metabolic activity assays, biofilm biomass quantification, and biofilm inhibition assays, depending on the methodology used in each study.

### Risk of bias evaluation

The risk of bias was assessed independently by two reviewers using the Joanna Briggs Institute (JBI) Critical Appraisal Checklist adapted for in vitro experimental studies. The assessment evaluated the clarity of study objectives, appropriateness of the experimental design, presence of comparator groups, standardization of antimicrobial testing methods, reliability of outcome measurements, and appropriateness of statistical analyses. Each domain was rated as “Yes,” “No,” “Unclear,” or “Not Applicable.” Studies with ≥80% “Yes” responses were classified as having a low risk of bias, 50–79% as moderate risk, and <50% as high risk. Any disagreements between reviewers were resolved through discussion or consultation with a third reviewer. We acknowledge that no universally accepted risk-of-bias tool exists specifically for in vitro antimicrobial studies, and therefore an adapted JBI checklist was used as the most appropriate available methodological appraisal tool.

## Results

### Study selection

The systematic search across PubMed, Scopus, Cochrane, and WEB of Science identified 312 records. After removal of duplicates (n = 78), 234 studies were screened based on titles and abstracts. Of these, 198 studies were excluded because they did not meet the predefined inclusion criteria.

**Fig. 1:**
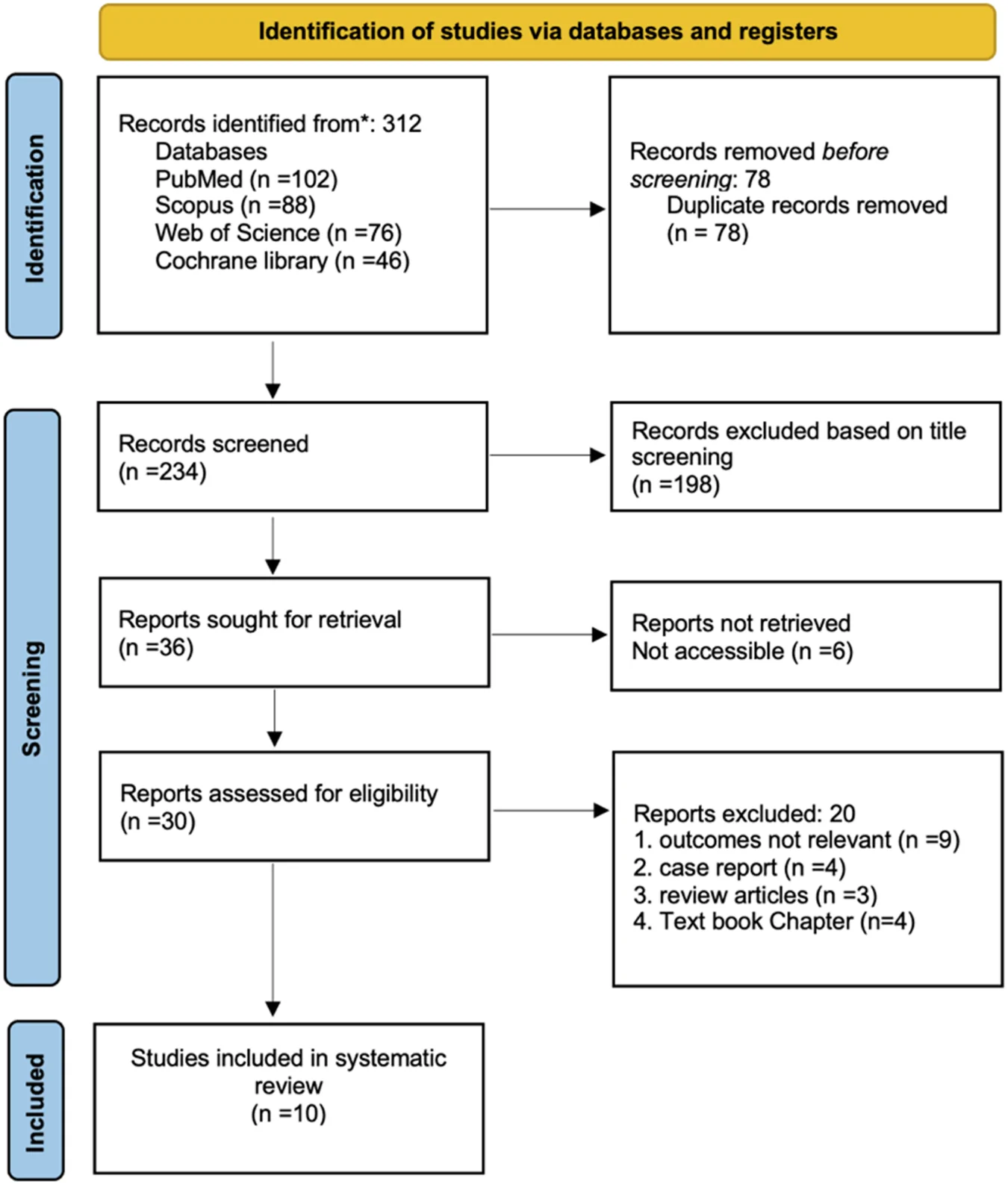
PRISMA 2020 flow diagram for new systematic reviews which included searches of databases and registers only

In total 36 full-text articles were screened to see if they met the criteria for inclusion; of these, 26 studies were excluded mainly because there were no proper comparator groups, there were no periodontal pathogens, or methodologic details were lacking. Therefore, ten in vitro studies were found to meet the criteria for inclusion and will be used in the qualitative synthesis. Figure 1.

### Included studies characteristics

Overall, the included studies demonstrated considerable methodological diversity. Porphyromonas gingivalis was the most frequently investigated periodontal pathogen, followed by *Aggregatibacter actinomycetemcomitans*, while several studies also evaluated other periodontopathogenic microorganisms, including *Fusobacterium nucleatum, Prevotella intermedia*, and *Tannerella forsythia*. The studies investigated both Ag and ZnO nanoparticles, with variations in particle size, morphology, synthesis methods, and concentrations. Antimicrobial efficacy was assessed using both planktonic and biofilm-based experimental methods. Planktonic antimicrobial assays included agar diffusion (zone of inhibition), minimum inhibitory concentration (MIC), and minimum bactericidal concentration (MBC). Biofilm-related outcomes were evaluated using colony-forming unit (CFU) enumeration, bacterial viability assays, metabolic activity assays, biofilm biomass quantification, and biofilm inhibition assays, depending on the methodology adopted in each study. Considerable methodological heterogeneity was observed across studies because different assays measured distinct aspects of antimicrobial activity.

Despite methodological differences, most studies reported positive antimicrobial activity of Ag and ZnO nanoparticles against periodontal pathogens, although the magnitude of the effect varied according to nanoparticle characteristics and experimental conditions. The details of the studies are summarized in Table 1.

**Table 1:** Summary characteristics

Author	Year	Microorganism(s) Evaluated	Nanomaterial Type	Comparison Agent	Method Used	Outcome Measure	Key Findings
Panpaliya et al.	[30]	*P. gingivalis*,	Ag nanoparticles	Chlorhexidine	Agar diffusion	Zone of inhibition	AgNPs showed comparable or higher inhibition than chlorhexidine
Zorraquín-Peña et al.	[35]	Periodontal bacteria	Ag nanoparticles	Chlorhexidine	MIC/MBC assay	MIC, MBC	Strong antibacterial activity with dose-dependent effect
Niska et al.	[31]	Oral pathogens	Ag nanoparticles	Chlorhexidine	MIC assay	MIC	Antimicrobial efficacy dependent on nanoparticle coating
Myronov et al.	[34]	Mixed bacteria	Ag nanoparticles	Chlorhexidine	Agar diffusion	Zone of inhibition	Combination showed synergistic antibacterial effect
Wang et al.	[33]	*P. gingivalis*, *A. naeslundii*	ZnOnanoparticles	Tetracycline	Growth inhibition assay	Bacterial viability	ZnO inhibited bacterial growth significantly
Ting et al.	[38]	Peri-implant pathogens	ZnO nanoparticles	Tetracycline	MIC assay	MIC	ZnO showed dose-dependent antibacterial activity
Al-Nema et al.	[37]	Oral pathogens	ZnO nanoparticles	Chlorhexidine	Agar diffusion	Zone of inhibition	ZnO exhibited moderate antimicrobial activity
Griauzdyte et al.	[36]	Periodontal pathogens	ZnO nanoparticles	Tetracycline	Systematic *in-vitro* analysis	MIC/MBC	ZnO effective but less potent than AgNPs
Deng et al.	[32]	Bacterial strains	Ag nanoparticles	Antibiotics (tetracycline)	MIC assay	MIC reduction	AgNPs enhanced antibiotic efficacy (synergism)
Martinez-Gutierrez et al.	[29]	Biofilm bacteria	Ag nanoparticles	Chlorhexidine	Biofilm assay	Biofilm inhibition	AgNPs disrupted biofilms more effectively

Considerable variability was observed in the concentrations of both nanoparticles and comparator antimicrobial agents across the included studies. Silver and Zinc oxide nanoparticles were evaluated over a wide range of concentrations, while comparator agents, including chlorhexidine, tetracycline, and other antibiotics, were also tested using different concentrations and formulations. Consequently, the antimicrobial efficacy reported in individual studies should be interpreted within the context of the specific concentrations investigated, as concentration-dependent effects were consistently observed. Direct comparison of antimicrobial outcomes across studies should therefore be undertaken with caution because differences in experimental concentrations may substantially influence the observed antimicrobial activity.

### Quality assessment

All included studies demonstrated clear objectives, appropriate methodologies, and reliable outcome measurements. The majority also incorporated control groups and appropriate analytical methods. Overall, most studies (8 out of 10) were assessed as having a low risk of bias, while two studies showed a moderate risk, primarily due to the absence of a control group or minor methodological limitations Table 2.

**Table 2:** Risk of bias assessment (JBI Format)

Study	Clear Objective	Appropriate Methodology	Control Group Present	Reliable Outcome Measurement	Appropriate Analysis	Overall Risk of Bias
Panpaliya et al. [30]	Yes	Yes	Yes	Yes	Yes	Low
Zorraquín-Peña et al. [35]	Yes	Yes	Yes	Yes	Yes	Low
Niska et al. [31]	Yes	Yes	Yes	Yes	Yes	Low
Myronov et al. [34]	Yes	Yes	Yes	Yes	Yes	Low
Wang et al. [33]	Yes	Yes	Yes	Yes	Yes	Low
Ting et al. [38]	Yes	Yes	Yes	Yes	Yes	Low
Al-Nema et al. [37]	Yes	Yes	Yes	Yes	Yes	Moderate
Griauzdyte et al. [36]	Yes	Yes	No	Yes	Yes	Moderate
Deng et al. [32]	Yes	Yes	Yes	Yes	Yes	Low
Martinez-Gutierrez et al. [29]	Yes	Yes	Yes	Yes	Yes	Low

### Antimicrobial efficacy

Due to heterogeneity in study designs, nanoparticle characteristics, and outcome measures, a meta-analysis was not feasible. However, a vote-counting and direction-of-effect synthesis was performed. Overall, Ag nanoparticles demonstrated superior antimicrobial efficacy compared to Chlorhexidine in 66.7% of studies, with the remaining showing comparable effects. Zinc oxide nanoparticles exhibited mostly comparable performance against chlorhexidine (66.7%), with limited superiority. When compared to Tetracycline, ZnO-NPs showed variable outcomes, with equal proportions of superiority, comparability, and inferiority. Notably, combinations of AgNPs with antibiotics consistently demonstrated superior antimicrobial efficacy (100%) over antibiotics alone Table 3.

**Table 3:** Overall antimicrobial superiority

Comparison	No. of Studies	Nanomaterials Superior	Comparable	Inferior
AgNPs vs Chlorhexidine	6	4 (66.7%)	2 (33.3%)	0
ZnO-NPs vs Chlorhexidine	3	1 (33.3%)	2 (66.7%)	0
ZnO-NPs vs Tetracycline	3	1 (33.3%)	1 (33.3%)	1 (33.3%)
AgNPs + Antibiotics vs Antibiotics	2	2 (100%)	0	0

Ag nanoparticles showed moderate to large antimicrobial effects with larger zones of inhibition and lower minimum inhibitory concentrations (MIC) than Zinc oxide nanoparticles. Zinc oxide nanoparticles had moderate effects and a strong dose-related response, but were generally less effective than AgNPs. Combinations containing AgNPs also had a synergistic effect that significantly lowered the MIC.

The majority of the studies included in this review had a low risk of bias (8 studies), and a minority of studies had a moderate risk (2 studies). It should be noted that none of the included studies were judged to have a high risk of bias, indicating that there is an overall acceptable methodological quality across the totality of the evidence included in this review. Master table included giving the comparative study about this survey and recently published review articles on the same or related fields Table 4.

**Table 4:** Comparison of the present systematic review with recently published systematic and narrative reviews on nanoparticles and local antimicrobial delivery in periodontal therapy

Study	Year	Review type	Primary focus	Nanomaterials / Interventions	Comparator / Delivery	Microorganisms / Outcomes	Key findings	Limitation / Gap addressed by present review
Yin et al. – The antibacterial mechanism of silver nanoparticles and its application in dentistry	[12]	Narrative review	Antibacterial mechanisms and dental applications	AgNPs	Dental applications; drug delivery discussed	Oral microorganisms; antimicrobial mechanisms	Discussed membrane disruption, ROS generation, Ag+ release and intracellular effects of AgNPs.	Focused primarily on AgNP mechanisms and dentistry; did not systematically compare AgNPs and ZnO NPs against periodontal pathogens.
Wei et al. – Local drug delivery systems as therapeutic strategies against periodontitis	[5]	Systematic review	Local drug delivery in periodontitis	Broad range of local antimicrobial/drug-delivery systems	Local delivery systems	Periodontal disease; clinical and experimental outcomes	Evaluated local delivery approaches and highlighted the potential benefits of localized antimicrobial delivery.	Broad local drug-delivery focus; not specifically a comparative evaluation of Ag and ZnO nanoparticles against conventional antimicrobials.
Steckiewicz et al. – Silver Nanoparticles as Chlorhexidine and Metronidazole Drug Delivery Platforms	[15]	Review-type article	AgNPs as antimicrobial drug-delivery platforms	AgNPs; AgNP–CHX and AgNP–metronidazole systems	Chlorhexidine and metronidazole delivery	Periodontal pathogens; antimicrobial activity and delivery potential	Highlighted the potential of AgNPs as platforms for delivery of conventional antimicrobials.	Primarily focused on AgNP-based drug delivery rather than systematically comparing AgNPs and ZnO NPs with conventional agents.
Griauzdyte & Jagelaviciene – Antimicrobial Activity of Zinc against Periodontal Pathogens	[36]	Systematic review	Antimicrobial activity of zinc against periodontal pathogens	Zinc compounds/materials, including zinc-based nanoparticles	Various zinc formulations	P. gingivalis, T. forsythia, T. denticola, P. intermedia, A. actinomycetemcomitans; antimicrobial and cytotoxic outcomes	Systematically assessed zinc activity against periodontal pathogens and considered cytotoxic effects on periodontal cells.	Focused on zinc rather than a direct AgNP-versus-ZnO NP comparison and comparison with CHX/tetracycline-based agents.
Amato et al. – Local Delivery and Controlled Release Drug Systems: A New Approach for the Clinical Treatment of Periodontitis	[14]	Narrative review	Local and controlled drug delivery in periodontitis	Nanoparticles, hydrogels, membranes and other delivery platforms	Local/controlled-release systems	Periodontal treatment and drug-delivery outcomes	Reviewed emerging controlled-release approaches and their potential clinical applications.	Broad delivery focus; did not specifically synthesize Ag and ZnO nanoparticle antimicrobial efficacy against periodontal pathogens.
Wang et al. – Nanoparticles in Periodontitis Therapy: A Review of the Current Situation	[4]	Narrative review	Comprehensive application of nanoparticles in periodontitis	Inorganic NPs, organic NPs and nanocomposites, including metal/metal oxide NPs	Nanocomposites, hydrogels, nanofibers, membranes and other delivery systems	Provided a broad overview of nanoparticles in periodontitis and discussed challenges and future directions.	Broad nanoparticle review; does not specifically provide a systematic comparative synthesis of Ag and ZnO versus CHX and tetracycline-based agents.	
Nakajima et al. – Advances in Local Drug Delivery for Periodontal Treatment: Present Strategies and Future Directions	[28]	Narrative review	Current and future local drug delivery strategies	Multiple nanomaterial and biomaterial-based systems	Gels, membranes, particles and other local delivery approaches	Periodontal therapy and translational/clinical considerations	Discussed advances in local delivery and future directions for periodontal treatment.	Primarily concerned with local drug delivery rather than comparative antimicrobial efficacy of AgNPs and ZnO NPs.
Present systematic review	2026	Systematic review	Comparative antimicrobial potential of Ag and ZnO nanomaterials against periodontal pathogens	AgNPs and ZnO NPs as separate interventions	CHX and tetracycline-based antimicrobial/local delivery agents	Periodontal pathogens; MIC, MBC, ZOI and biofilm-related outcomes	Synthesizes available in-vitro evidence comparing AgNPs and ZnO NPs with conventional antimicrobial agents while considering nanoparticle characteristics, concentrations and methodological heterogeneity.	Addresses the comparative evidence gap while critically considering concentration, nanoparticle characteristics, assay methods and biological safety.

In summary, the synthesis of in vitro evidence indicates that Ag nanoparticles are significantly more effective at preventing the growth of periodontal pathogens than Zinc oxide nanoparticles; however, combination strategies will likely lead to the greatest overall improvements in antimicrobial activity.

## Discussion

A systematic review was conducted in order to evaluate and compare antimicrobial effectiveness between nanomaterials containing Ag, or ZnO to conventional antimicrobial agents such as chlorhexidine and tetracycline -based local drug delivery systems against common periodontal pathogens using in vitro research methods [22–24]. Because microbial dysbiosis (unbalanced microorganisms) within the subgingival biofilm is the primary driver of periodontal disease, antimicrobial strategies represent an important therapeutic component for treating periodontal disease [25]. Results of studies included in the present review indicate that nanomaterials, specifically Ag and ZnO nanoparticles, have significant antimicrobial activity against key peri-odontal pathogens [25–27].

Extensive reports demonstrate Ag nanoparticles to have considerable antimicrobial activity due to their ability to attach to bacterial membranes, disrupt cellular integrity and metabolism. The large ratio of surface area to volume of nanoparticles allows for greater reactivity and allows for the effective interaction between nanomaterials and microorganisms [28, 29]. In summary, results of recent studies indicated significant antimicrobial effects of Ag nanoparticles against common periodontal pathogens including *Porphyromonas gingivalis and Aggregatibacter actinomycetemcomitans*; moreover, Ag nanoparticles demonstrated comparable or greater antimicrobial activity as compared with traditional antimicrobial agents like chlorhexidine or tetracycline [30].

The included reports show that Zinc oxide nanoparticles (ZnO-NPs) are able to effectively kill microbes. The mechanisms by which ZnO-NPs kill bacteria are by producing oxyhalides, disrupting membranes of cells, and releasing zinc ions into the environment which interferes with cellular actions. The antimicrobial ability of ZnO-NPs was influenced by variables such as size of the particles, concentration of the particles, how they were produced and experimental conditions, with most of the studies finding smaller ZnO-NPs and higher concentrations had a greater ability to kill microbes. The results of studies that incorporated ZnO-NPs compared to traditional agents were variable. The results from multiple studies relative to chlorhexidine or tetracycline showed a number of studies reporting similarly or better antimicrobial action of ZnO-NPs, however some studies reported similar actions to these agents. It is difficult to directly compare results from different studies because of the use of different experimental methods, different bacterial strains and different methods of evaluating results (e.g., minimum inhibitory concentration, minimum bactericidal concentration and zone of inhibition) [31, 32].

An important consideration when interpreting the findings of this review is the marked variation in nanoparticle and comparator concentrations used across studies. Antimicrobial activity of both Ag and ZnO nanoparticles was frequently concentration-dependent, with higher concentrations generally producing greater bacterial inhibition or lower MIC values. Likewise, the effectiveness of comparator agents depended on the concentration and formulation employed. Therefore, statements regarding the superiority or comparability of nanoparticles should be interpreted within the experimental context of each study rather than generalized across all investigations. This variability further contributed to methodological heterogeneity and limited the feasibility of quantitative evidence synthesis.

One important source of heterogeneity among the included studies was the diversity of antimicrobial assessment methods. While several studies evaluated planktonic bacterial susceptibility using minimum inhibitory concentration (MIC), minimum bactericidal concentration (MBC), or agar diffusion assays, others investigated antimicrobial efficacy against established biofilms using colony-forming unit (CFU) enumeration, bacterial viability assays, metabolic activity assays, biofilm biomass quantification, or biofilm inhibition assays. Because these methodologies assess different biological endpoints, direct quantitative comparisons between studies were not feasible, thereby supporting the decision to perform a narrative synthesis rather than a meta-analysis.

Nanomaterials are promising adjuncts in periodontal therapy because of their antimicrobial properties and potential for controlled local drug delivery. Although previous studies suggest that nanoparticles may enhance biofilm penetration due to their small size and large surface area, only a limited number of studies in this review directly evaluated biofilm-related outcomes; therefore, this should be considered a proposed mechanism rather than a definitive finding.

Silver and Zinc oxide nanoparticles exert antimicrobial effects through multiple mechanisms, including membrane disruption, reactive oxygen species generation, metal ion release, and interference with bacterial cellular functions, which may reduce the likelihood of antimicrobial resistance compared with conventional antibiotics, although resistance to nanoparticles has also been reported. However, their biological safety remains an important consideration, as cytotoxicity depends on factors such as nanoparticle composition, size, concentration, surface characteristics, and exposure duration. Therefore, further standardized in vitro, in vivo, and clinical studies are required to establish their long-term safety, optimal therapeutic concentrations, and clinical applicability in periodontal therapy [33].

Recent studies demonstrating the antimicrobial activity of Ag and ZnO nanomaterials against oral and periodontopathic bacteria confirm the findings of this current systematic review. Myronov P and colleagues (2021), for example, found that combining chlorhexidine with Ag nanoparticles produced a greater degree of bacterial killing and improved wound healing compared to chlorhexidine alone. This synergistic relationship supports the findings of this review because Ag nanoparticles often had comparable or superior action to traditional antimicrobial agents. Furthermore, the ability of Ag nanoparticles to augment the effect of traditional antimicrobial agents on oral microorganisms further supports their use in combination as adjunctive therapeutic agents in periodontal treatment [34].

Likewise, Zorraquín-Peña I and colleagues (2020) indicated that Ag nanoparticles with glutathione inhibit the growth of specific oral bacteria causing periodontal disease and have also been assessed for toxicity and inflammatory effects to oral cells. This finding supports the conclusion found within the current review that Ag nanoparticles possess strong antimicrobial properties that are most effective against the major pathogenic microorganisms of periodontal disease, especially Porphyromonas gingivalis. However, the authors of this study also noted that there were major deficiencies in how many of the in vitro studies referenced in this review addressed the issue of biocompatibility; thus, indicating a substantial area needing further investigation [35].

In the related systematic review identified by Griauzdyte V and Jagelaviciene E (2023), zinc was found to exert a large effect on the viability of bacteria associated with periodontal disease. However, the activity of zinc against these bacteria varies depending on the type of zinc used as well as the conditions under which it is tested. This is consistent with our own observations in which we found that ZnO nanoparticles had variable antimicrobial activity in vitro and less consistent levels of activity than Ag nanoparticles. When considering the antimicrobial action of either of these materials, it is critical to consider particle size, concentration and method of synthesis, which all play an important role in terms of the antimicrobial properties of ZnO or Ag nanomaterials [36].

In addition, Al- Nema Hamzah et al. [37] evaluated antimicrobial effects exhibited by using Zinc oxide nanoparticles mixed into alginate impression making materials. The significance of this study provides a translational perspective as it shows where implementation of Zinc oxide nanoparticles in dental materials can yield antimicrobial effects. Although this review focused primarily on direct comparisons for antimicrobial activity, these types of implementations highlight the larger clinical uses of nanomaterials outside traditional delivery methods for medication [37].

Moreover, Ting BY and others (2022) explored biosynthesized ZnO nanoparticles and discovered that they show significant antimicrobial activity against peri-implantitis-associated pathogens. Therefore, their results half support the conclusion of this review paper; that is, that ZnO nanoparticles possess excellent antibacterial properties, especially when using biologically optimized biosynthetic techniques. Finally, this research highlights the importance of how nanoparticles are synthesized, as this can significantly affect antimicrobial effectiveness. This may also be responsible for the variability found in the antimicrobial effectiveness results from the studies included in this review [38].

In summary, the current literature shows support for this systematic review’s findings regarding the greater consistency and stronger antimicrobial activity of Ag nanoparticles compared with ZnO nanoparticles, which have been shown in previous studies to have promising but inconsistent antimicrobial activity. Furthermore, these studies suggest that the use of new trends such as synergistic combinations, use in conjunction with biomaterials, and consideration of biocompatibility and how they were synthesized were inconsistently reported in all the in vitro studies included in this review. The findings thus provide additional support for the need for standardization of research methodologies and further translation and clinical evaluation studies to confirm the efficacy of using nanomaterials for periodontal treatment [39, 40].

Silver and zinc oxide nanoparticles have potential applications as antimicrobial drug-delivery platforms in periodontal therapy. Their small size, high surface area, and ability to interact with microbial cells may facilitate localized delivery and sustained release of antimicrobial agents within periodontal pockets. Nanoparticle-based systems may therefore enhance local antimicrobial activity while reducing systemic exposure. However, the optimal nanoparticle formulation, drug-loading capacity, release characteristics, therapeutic concentration, and biological safety remain insufficiently established. Further standardized preclinical and clinical studies are required to determine whether these systems can provide clinically meaningful advantages over conventional local antimicrobial delivery approaches.

This systematic review differs from previous reviews by directly comparing silver and Zinc oxide nanoparticles with chlorhexidine and tetracycline against periodontal pathogens. It included only comparative in vitro studies, critically appraised methodological quality, synthesized evidence from planktonic and biofilm-based assays, and identified methodological limitations and translational challenges, providing direction for standardized preclinical and clinical research.

Although Ag and ZnO nanoparticles demonstrated promising antimicrobial activity, their biological safety requires careful evaluation. Factors such as cytotoxicity, biocompatibility, genotoxicity, and long-term tissue responses were not consistently assessed in the included studies, limiting conclusions regarding their clinical safety.

This review has several limitations. Considerable heterogeneity in nanoparticle characteristics (type, size, morphology, synthesis method, and concentration), comparator agents, microorganisms, and antimicrobial assessment methods limited direct comparison across studies and precluded meta-analysis. Furthermore, only a limited number of studies evaluated biofilm-based outcomes, with most relying on planktonic assays. There is also no current consensus regarding the optimal nanoparticle formulation, drug delivery system, or administration method for periodontal therapy. Future standardized in vitro, in vivo, and clinical studies are needed to facilitate meaningful comparisons and clinical translation. Another limitation was the inconsistent reporting and wide variation of nanoparticle and comparator concentrations among the included studies.

Future research should focus on standardized biological evaluations of silver and Zinc oxide nanoparticles, including cytotoxicity, biocompatibility, genotoxicity, and long-term safety assessments. In addition, translational studies evaluating optimal formulations, drug delivery systems, therapeutic concentrations, and clinical efficacy are needed to support the development of safe and effective nanoparticle-based products for periodontal therapy.

## Conclusion

Silver and Zinc oxide nanoparticles demonstrate promising antimicrobial potential against periodontal pathogens; however, the current evidence is limited by methodological heterogeneity and is largely based on in vitro studies. Before clinical application, comprehensive evaluation of the biological and physicochemical properties of nanoparticle-incorporated formulations, including gels, local drug delivery systems, and other biomaterial-based platforms, is essential to establish their safety, stability, and therapeutic efficacy. Well-designed translational studies and clinical trials are required to support the development of safe and effective nanoparticle-based therapies for periodontal applications.

## Data Availability

No datasets were generated or analysed during the current study.
